# A comparative study on the equine and camelid antivenoms upon cardiovascular changes induced with *Hemiscorpius lepturus* venom in rats

**DOI:** 10.22038/IJBMS.2019.14052

**Published:** 2019-12

**Authors:** Hossein Fatemikia, Mostafa Kamyab, Ali Movahed, Mehdi Sadeghi, Euikyung Kim, Mahdi Behdani, Naser Mohammadpour Dounighi, Mehrnaz Shahrivar, Ramin Seyedian

**Affiliations:** 1 Department of Physiology, School of Medicine, Shiraz University of Medical Sciences, Shiraz, Iran; 2 Department of Aquatic Biotechnology, Faculty of Life Sciences and Biotechnology, Shahid Beheshti University, Tehran, Iran; 3 The Persian Gulf Tropical Research Center, Biochemistry Group, Bushehr University of Medical Sciences, Bushehr, Iran; 4 Department of Physiology, School of Medicine, Bushehr University of Medical Sciences, Bushehr, Iran; 5 College of Veterinary Medicine, Gyeongsang National University, Jinju, South Korea; 6 Biotechnology Research Center, Venom and Biotherapeutics Molecules Laboratory, Pasteur Institute of Iran, Tehran, Iran; 7 Department of Human Vaccine and Serum, Razi Vaccine and Serum Research Institute, Agricultural Research, Education and Extension Organization, Karaj, Iran; 8 School of Medicine, Bushehr University of Medical Sciences, Bushehr, Iran; 9 Department of Pharmacology, Bushehr University of Medical Sciences, Bushehr, Iran

**Keywords:** Camelid antivenom, Cardiovascular, Envenomation, Equine antivenom, Hemiscorpius lepturus

## Abstract

**Objective(s):**

In this study, the neutralizing abilities of the equine and the recently introduced camelid antivenoms on the hemodynamic parameters (inotropism, chronotropism, and arrhythmogenicity) were assessed following envenomation by *Hemiscorpius lepturus* venom in rats.

**Materials and Methods:**

At first, the electrophoretic profiles of both products were obtained by using the SDS-PAGE method (12.5%) and stained with Coomassie blue and silver nitrate. Secondly, different doses of the camelid antivenom (10, 50, and 100 µl) were given intravenously in 10 min before venom injection (400 µg/rat). The neutralizing potencies of camelid and equine antivenoms were measured by preincubation (100 µl) with *H. lepturus *venom for 30 min at room temperature. Finally, equal amounts of the antivenoms were injected intravenously to observe the hemodynamic changes.

**Results:**

Based on the electrophoretic profile, it was evident that undesired proteins significantly decreased in equine antivenom, owing to impurities. Pretreatment with the camelid antivenom (100 µl), neutralized the elevation of the mean arterial pressure evoked with scorpion venom injection (88.15±4.56 versus 10.2±1.23 percent at the 8th min). The Incubation of the venom and the camelid antivenom counteracted the hemodynamic changes, but the equine product had no effect. The intravascular injection of the equine antivenom transiently increased the mean arterial pressure as compared to the control (108.67±8.63 mmHg versus 52.67±1.93 mmHg at the 10^th^ min).

**Conclusion:**

The most obvious finding emerging from this study was that the camelid antivenom neutralized the hemodynamic changes in rats significantly, but in comparison, the equine antivenom had just a minor ability.

## Introduction

Scorpion sting as the oldest arthropod (20 families, 208 genera, and 2231 species) (1) is common in the tropical and subtropical areas of the world, including Iran, especially in south-western regions (2). To date, various research has tended to reveal many detrimental cardiovascular changes including hypertension, pulmonary edema, and arrhythmia in the animals due to envenomation (3, 4). In this regard, a strong relationship has been found between the initial stimulatory effects of the venom on the autonomic nervous system and the subsequent cardiac changes (4).

It is known that *Hemiscorpius lepturus* is one of the most dangerous scorpions in Iran (5). In most cases, the clinical symptoms of the envenomed patients (hematuria, cardiovascular deteriorations, and dermal lesions) are not serious, while in a minority of cases, especially in infants, it will lead to renal tubular necrosis and death due to hemolytic uremic syndrome (6). 

The treatment of scorpion sting needs symptomatic therapy and the injection of equine polyvalent antivenom, which has been produced against six dangerous Iranian scorpions*. *There is controversy over the effectiveness of the conventional equine antivenom since Fab and F (ab′)^2^ products reach the vital organs 5 to 9 times later following the venom exposure because of their large sizes (7, 8). Moreover, early or late anaphylactic reactions may be observed in victims (9%) treated with the medication (9). However, camelid antivenom has been introduced recently as a novel serum therapy against snake and scorpion envenomations due to its unique properties. Furthermore, the absence of the light chains and the first part of the heavy chain in the serum of this animal makes it more compatible with humans. Besides, the adverse anaphylactic reactions are reduced due to the compatibility of its Ig G content (10). Therefore, this experimental animal study set out to compare the neutralizing effects of the camelid and equine antivenoms upon transient hypertension and bradycardia caused by the venom. Ultimately, it evaluates the hemodynamic changes induced by their own injections (11).

## Materials and Methods

***Venom and antivenom preparation***

*H. lepturus* crude venom and the equine antivenom were obtained from Razi Institute of Iran (Karaj province). The precipitated camelid antivenom with saturated ammonium sulfate (SAS) was taken from Iranian Pasteur Institute following immunization of young camels with *H. lepturus* venom (12).

In this case, raw venoms were milked by applying direct electrical shock (15 V) to their telsons. The collected venom was transferred to a microcentrifuge tube, lyophilized, and stored at -20 ^°^C following centrifugation at 1000 rpm for 20 min. The polyvalent antivenoms (5 ml ampoules), were used in our study. 

***SDS-PAGE***

At first, the protein concentrations of our samples were measured by the Bradford method with bovine serum albumin as the control (13). Secondly, the camelid and equine antivenom samples were heated at 95 °C for 5 min and then cooled on ice. The protein components of both products were analyzed using SDS-PAGE with 12.5 % gels according to the Laemmli method. Gels were stained with Coomassie blue R-250 and silver nitrate to visualize the protein bands. Protein quantitation was achieved using a prestained protein ladder (CSL-BBL Cleaver company), ranging from 11-245 kDa. 

***Experimental protocol***

Male Wistar rats (250–300 g) were housed in polycarbonate cages with free access to water and chow in the animal house of Bushehr University of Medical Sciences for one week before our study. They were anesthetized with ketamine (100 mg/kg, IP) and xylazine (10 mg/kg, IP) before the experiment.

The anesthetized rats were placed supine under a heat lamp, and their temperature was monitored with a rectal tube connected to a thermometer (Physitemp BAT-12, Texas Scientific Instruments, San Antonio, Texas, USA). An incision was made in the right femoral area, and two cannulas were inserted in the femoral artery and vein, in order to administer the venom/antivenom and measure hemodynamic changes (arterial pressure, heart rate, and arrhythmia). Our data were analyzed using a pressure transducer (MLT844, AD Instruments, Australia) for a sustained recording of the arterial pressure by means of a Power Lab/4SP data acquisition system (AD Instruments). Final results were obtained 20 min after the beginning of the experiments following the administration of the venom or antivenoms. Mean arterial pressure was calculated using the following formula:  [(2 x diastolic) + systolic] divided by 3.

***Antivenom effects***

Three groups of rats (six animals each) were pretreated with different doses of the camelid antivenom (10, 50, and 100 µl in a dose of 200 µl of saline) slowly ten min before the venom injection (400 µg/rat) (11). The last group was treated with normal saline, and the study was continued. In another set of our experiments, two doses of the camelid and equine antivenoms (100 µl) were mixed with the venom and incubated half an hour at room temperature. The hemodynamic parameters (inotropism, chronotropism, and arrhythmogenicity) were measured following the intravenous injection of the cocktail.

***The effects of the camelid and equine antivenoms on hemodynamic parameters***

The animals were divided into three groups (n=6), and the cardiac parameters were measured 20 min before the antivenin medications. Normal saline (200 µl) was injected in the first group as a negative control. The camelid and equine antivenoms with the same volume were infused via the femoral vein in two minutes. Inotropic, chronotropic, and arrhythmogenic parameters were evaluated in the last two groups and compared with the former. 

***Statistical analysis***

Results were expressed as mean±SD (standard deviation of the mean) and were evaluated using one- or two-way analysis of variance (ANOVA) followed by Tukey’s *post hoc* test. Results were also analyzed by Student’s t-test. In all cases, differences were considered significant at *P-*value<0.05

## Results

***Gel electrophoresis***

**Figure 1:**
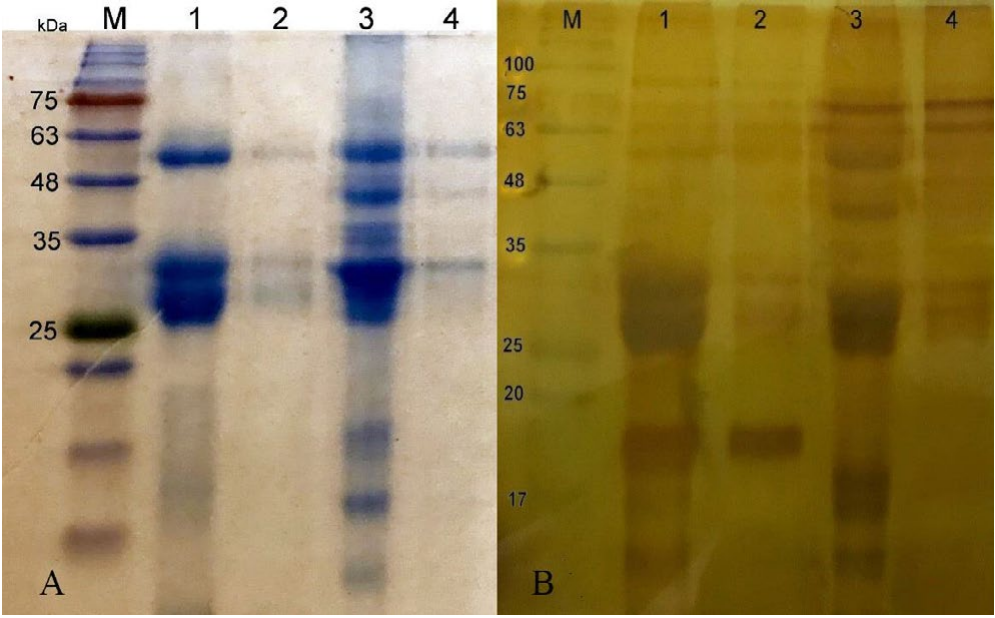
SDS- PAGE analysis of the camelid and equine antivenoms. Lanes 1 and 2 are the equine antivenoms, while lanes 3 and 4 are the camelids in Coomassie (A) and silver nitrate staining (B). M is the protein marker ranging from 11-245 kDa

The electrophoretic profiles of the camelid and conventional antivenoms showed at least 7 and 3 bands in Coomassie blue staining distributed between 11-260 kDa (Figure 1A). The major bands of these two products were located at 59, 46, 36, 32, 18, and 14 kDa and 61, 33, and 19 kDa, respectively. The other protein bands were revealed after silver nitrate staining in both products (Figure 1B). 

***Neutralizing effects of the camelid antivenom***

**Figure 2:**
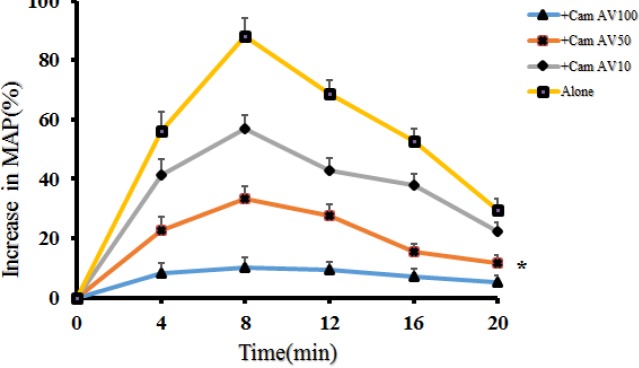
The effects of escalating doses of camelid antivenom (10, 50, and 100 µl) on inotropic responses in anesthetized rats.* P-value<0.01, significantly different from the venom injection, analyzed with repeated measures ANOVA test

The scorpion venom evoked transient hypertensive effects considerably, and it reached the maximum level of 88.15% increase in mean arterial pressure (MAP) in eight minutes (Figure 2). The prior instillation of the camelid antivenom (100 µl) significantly reduced this property (10.24 versus 88.15 percent). This volume was selected for our later experiments. 

***Hemodynamic potencies of the camelid and equine antivenoms***

**Figure 3:**
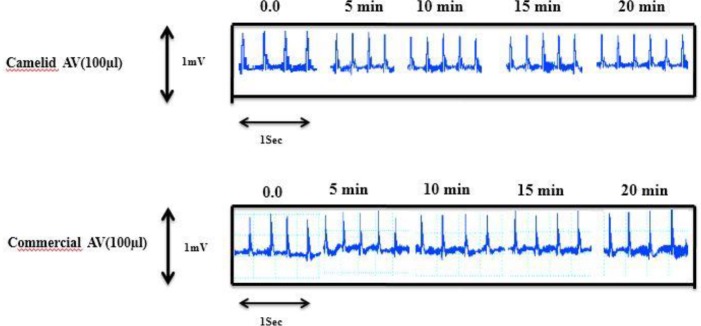
Arrhythmogenic evaluation of the camelid and equine antivenom in rats. There was no evidence of arrhythmogenicity in either trace upon time

Injection of the camelid and equine antivenoms (100 µl/rat; IV) in separate experiments caused different inotropic, chronotropic, and arrhythmogenic properties among the anesthetized rats. There was no increase in MAP within 20 min after the camelid antivenom injection as compared to normal saline (Table 1), while it stands out that there was a significant difference in MAP between the equine and control during this time. Despite the significant bradycardia caused by the equine injection, there were no alterations in the camelid product (data not shown). According to Figure 3, there was, however, no evidence of arrhythmogenicity in either group during this procedure.

***Neutralizing efficiency of the incubated products***

**Figure 4:**
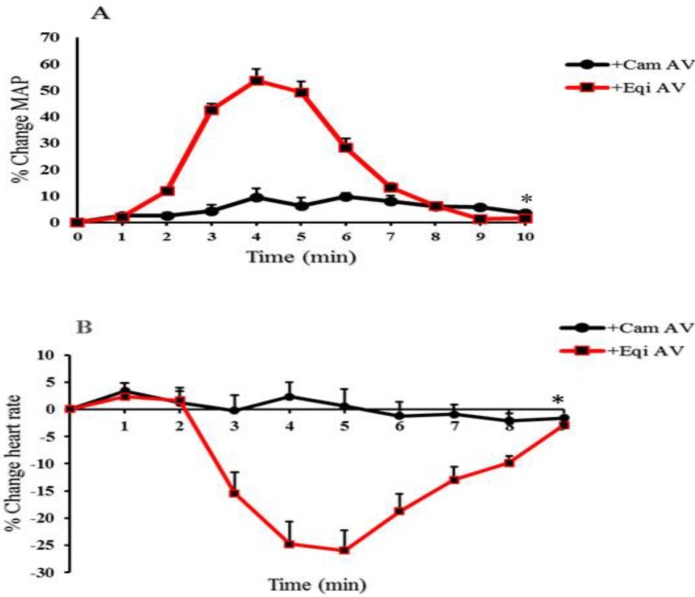
Inotropic and chronotropic effects (Figures 4A and 4B) of *H. lepturus* venom (400 µg/rat) incubated with equine (Eqi AV) or camelid antivenoms (Cam AV) with an equal volume (100 µl; IV)

The scorpion venom incubated with the equine antivenom (100 µl) increased the mean arterial pressure 53.8% at the fourth minute in the treated rats (Figure 4A). Additionally, it had no neutralizing potencies upon the negative chronotropic changes since the value of the heart rate had transient decrease to 25.97% below the initial value five minutes after the intravascular injection (Figure 4B). No significant alterations were observed in the arterial pressure and the heart rate following incubation with the camelid antivenom.

**Table 1:** Mean arterial pressure changes upon time after camelid and equine antivenom injection in rats

Time (min)	Control	Camelid AV (100 μl)	Equine AV (100 μl)
0	67.33±2.21^a^	73.33±4.56	76.24±6.34
5	55.12±3.13	60.12±3.94	86.12±7.23^*^
10	52.67±1.93	62.34±5.12	108.67±8.63^*^
15	63.12±4.24	68.43±4.17	94.67±6.37^*^
20	59.33±3.76	66.67±3.29	81.33±5.78^*^

^a^ Values are the mean±SD of the mean arterial pressure (mm Hg) in each group (n=6) before and after intravenous injection of the camelid and equine antivenoms

* Significantly different from the control group, ANOVA test, *P*-value<0.05

*P-value<0.01, significantly different from the equine antivenom

## Discussion

Scorpions are considered a threat to farmers, villagers, and passengers, especially in dry and hot environments. It is estimated that approximately 2.3 billion people are prone to scorpion bite (14). However, the number of envenomations is greater than one million annually (15). It should be pointed out that Iran has a large fauna with at least 52 species of this animal among Middle East countries (16).

Prior studies have noted the importance of the careful evaluation of the hemodynamic changes, including myocardial damage, pulmonary edema, and occasional hypertension, due to scorpion envenomation, especially among infants (17). 

Cardiovascular deterioration caused by envenomation necessitates equine antivenom injection and symptomatic therapy as early as possible. Recently, camelid antivenom has been used due to its lower immunogenicity, more thermo-stability, and less potency to induce anaphylactic shock (18). 

It must be taken into account that *H. lepturus,* which belongs to the Scorpionidae family, has lower cardiogenic effects on rats compared to other venomous scorpions like *Tityus serrulatus *(1200 µg/kg versus 200 µg/kg) (11, 19-20). 

Furthermore, this study found that more attempts have been carried out by Razi Institute antivenom to eliminate the impurities of the equine antivenom (21). 

Further analysis showed that pretreatment with the camelid antivenom (100 µl) could significantly neutralize the transient arterial pressure elevation induced by* H. lepturus *venom (Figure 2). However, it had no neutralizing potency when injected soon after venom instillation (data not shown). According to our experimental results, it seems that contrary to the equine antivenom, heavy chain antibodies in the camelid product make it a suitable remedy due to its safety, high affinity, and finally cardiovascular neutralizing potency (22-25). More precautions should be taken to inject the equine antivenom intravenously since it can elevate mean arterial pressure by itself in a short time (Table 1). Unlike other antivenoms, the definite causes of the hypertensive property of this product are unknown and requires more investigation (26, 27). 

The present experiment was in line with the previous studies showing no neutralizing effects following animal pretreatment with the equine antivenom against cardiovascular changes caused by the venomous animals (3, 4, 28). Furthermore, the previous report has also shown the venom neutralizing capacity of the camelid antivenom against hemodynamic deterioration following *Hottentota saulcyi *envenomation (29).

## Conclusion

There is no consensus among scientists regarding the neutralizing potency of equine antivenom on hemodynamic changes following envenomation in animals and humans. Returning to the hemodynamic results obtained in this study, it is evident that Razi Institute antivenom should be slowly infused in envenomed rats owing to its own tendency to raise mean arterial pressure. Furthermore, the camelid antivenom could be introduced as a novel therapy counteracting the hemodynamic dramatic changes.

## References

[1] Dunlop JA, Selden PA (2013). Scorpion fragments from the Silurian of Powys, Wales. J Arachnol.

[2] Shahbazzadeh D, Amirkhani A, Djadid ND, Bigdeli S, Akbari A, Ahari H (2009). Epidemiological and clinical survey of scorpionism in Khuzestan province, Iran (2003). Toxicon.

[3] Gueron M, Ilia R, Sofer S (1992). The cardiovascular system after scorpion envenomation A review. J Toxicol Clin Toxico.

[4] Gueron M, Yaron R (1970). Cardiovascular manifestations of severe scorpion sting: clinicopathologic correlations. Chest.

[5] Jalali A, Rahim F (2014). Epidemiological review of scorpion envenomation in Iran. Iran J Pharmaceu Res.

[6] Valavi E, Ansari MA (2008). Hemolytic uremic syndrome following Hemiscorpius lepturus (scorpion) sting. Indian J Nephrol.

[7] Alirahimi E, Kazemi-Lomedasht F, Shahbazzadeh D, Habibi-Anbouhi M, Hosseininejad-Chafi M, Sotoudeh N (2018). Nanobodies as novel therapeutic agents in envenomation. BBA-General Subjects.

[8] Ismail M, Abd-Elsalam M (1998). Pharmacokinetics of 125I-labelled IgG, F (ab′) 2 and Fab fractions of scorpion and snake antivenins: merits and potential for therapeutic use. Toxicon.

[9] Freire-Maia L, Campos J, Amaral C (1994). Approaches to the treatment of scorpion envenoming. Toxicon.

[10] Cook DA, Samarasekara CL, Wagstaff SC, Kinne J, Wernery U, Harrison RA (2010). Analysis of camelid IgG for antivenom development: Immunoreactivity and preclinical neutralisation of venom-induced pathology by IgG subclasses, and the effect of heat treatment. Toxicon.

[11] Pourkhalili K, Fatemikia H, Kim E, Mashayekhy NR, Dounighi NM, Hajivandi A (2018). Hemodynamic changes in experimentally envenomed anaesthetized rats by intravenous injection of Hemiscorpius lepturus venom. J Arthropod-Borne Di.

[12] Behdani M, Zeinali S, Karimipour M, Shahreza HK, Ghasemi P, Asadzadeh N (2010). Antiserum production in immunized camel by the venom of Hemiscorpius lepturus scorpion: evaluation of neutralizing test in vivo. Tehran Univ Med J.

[13] Bradford MM (1976). A rapid and sensitive method for the quantitation of microgram quantities of protein utilizing the principle of protein-dye binding. Anal Biochem.

[14] Chippaux J-P, Goyffon M (2008). Epidemiology of scorpionism: a global appraisal. Acta Trop.

[15] Isbister GK, Bawaskar HS (2014). Scorpion envenomation. New England J Med.

[16] Dehghani R, Fathi B (2012). Scorpion sting in Iran: a review. Toxicon.

[17] Sofer S, Gueron M (1990). Vasodilators and hypertensive encephalopathy following scorpion envenomation in children. Chest.

[18] Cook DA, Owen T, Wagstaff SC, Kinne J, Wernery U, Harrison RA (2010). Analysis of camelid antibodies for antivenom development: Neutralisation of venom-induced pathology. Toxicon.

[19] Freire-Maia L, Pinto G, Franco I (1974). Mechanism of the cardiovascular effects produced by purified scorpion toxin in the rat. J Pharmacol Exp Ther.

[20] Pucca MB, Cerni FA, Junior ELP, Bordon KdCF, Amorim FG, Cordeiro FA (2015). Tityus serrulatus venom–a lethal cocktail. Toxicon.

[21] Seyedian R, Pipelzadeh MH, Jalali A, Kim E, Lee H, Kang C (2010). Enzymatic analysis of Hemiscorpius lepturus scorpion venom using zymography and venom-specific antivenin. Toxicon.

[22] Lauwereys M, Ghahroudi MA, Desmyter A, Kinne J, Hölzer W, De Genst E (1998). Potent enzyme inhibitors derived from dromedary heavy-chain antibodies. EMBO J.

[23] Lovreček D, Tomić S (2011). A century of antivenom. Collegium Antropol.

[24] Padula AM, Winkel KD (2016). Fatal presumed tiger snake (Notechis scutatus) envenomation in a cat with measurement of venom and antivenom concentration. Toxicon.

[25] Padula AM, Winkel KD (2017). Antivenom production in the alpaca (Vicugna pacos): Monovalent and polyvalent antivenom neutralisation of lethal and procoagulant toxins in Australian elapid venoms. Small Rumin Res.

[26] Ramasamy S, Isbister GK, Seymour JE, Hodgson WC (2004). The in vivo cardiovascular effects of box jellyfish Chironex fleckeri venom in rats: efficacy of pre-treatment with antivenom, verapamil and magnesium sulphate. Toxicon..

[27] Sofer S, Shahak E, Gueron M (1994). Scorpion envenomation and antivenom therapy. J Pediatr.

[28] Abroug F, ElAtrous S, Nouria S, Haguiga H, Touzi N, Bouchoucha S (1999). Serotherapy in scorpion envenomation: a randomised controlled trial. The Lancet.

[29] Darvish M, Ebrahimi SA, Shahbazzadeh D, Bagheri KP, Behdani M, Shokrgozar MA (2016). Camelid antivenom development and potential in vivo neutralization of Hottentotta saulcyi scorpion venom. Toxicon.

